# Gamified interventions to improve the knowledge, attitude and practice on rational use of antibiotics among school children in Mysuru, South India, to curb the growing antimicrobial resistance (AMR)

**DOI:** 10.3389/fpubh.2025.1574647

**Published:** 2025-09-02

**Authors:** Mahadevaiah Neelambike Sumana, Supreeta R. Shettar, Yogeesh D. Maheshwarappa, G. K. Megha, Veerabhadra Swamy G. S., Chinchana Shylaja Eshwarappa, Shruthi Shree S. C.

**Affiliations:** Department of Microbiology, JSS Medical College and Hospital, JSS Academy of Higher Education and Research, Mysuru, India

**Keywords:** antimicrobial resistance, fun-filled learning, gamified interventions, knowledge, awareness and practice on antibiotic use, rational use of antibiotics

## Abstract

**Background:**

Antimicrobial resistance (AMR) is a global emergency, and the general public and School children are limited in their awareness of the rational use of antibiotics. Gamified education can effectively address this issue. This study aimed to improve knowledge, attitudes, and practices regarding rational antibiotic use through innovative games.

**Methodology:**

A gamified educational intervention was conducted with 2,195 high school students (13–16 years) in Mysuru, South India. The session included a blackboard introduction to microbes and antibiotics, an animated video on irrational antibiotic use, and games like Bucketing the Ball and Monkeying with Donkey to teach when antibiotics are unnecessary (e.g., respiratory tract infections and gastroenteritis). Pre- and post-test questionnaires were administered and analysed.

**Results:**

Results showed significant improvements: knowledge of antibiotic effects increased from 2.5 to 82.5%; understanding of antibiotics for bacterial infections rose from 11.5 to 82.5%; awareness of when to avoid antibiotics for common infections improved from 5.1 to 96.77%; and awareness of their use for urinary, skin, and soft tissue infections increased from 19.6 to 90.38%. Practices like not buying antibiotics without a prescription and completing the course improved from 20.3 to 91.92%.

**Conclusion:**

Gamified education effectively enhances knowledge, awareness, and practice of rational antibiotic use. With rising AMR, such innovative interventions are crucial to educating the general public and School children, ensuring a long-lasting impact on this global issue.

## Introduction

Antimicrobial resistance (AMR) is one of the most alarming problems and toughest challenges posed to human health this century (1–3). As the AMR crisis reaches its peak, experts have declared that the era of ineffective antibiotics is fast approaching (4). The World Health Organisation (WHO) estimates that about half of antimicrobial medicines are inappropriately prescribed, and roughly half of patients fail to complete their prescribed courses (5). The clinical efficacy of antibiotics greatly depends on their correct use, which, in turn, is influenced by patients, physicians, and retailers (5). A lack of knowledge, along with widespread issues in attitudes, beliefs, and behaviours, has been reported among consumers, directly impacting rational antibiotic use (5).

The lack of knowledge and widespread problems in attitudes, beliefs, and behaviours among consumers directly influence rational antibiotic usage (5). Physicians’ decisions are also influenced by several factors, such as the fear of losing patients’ trust, insufficient information on indications for antibiotic use, and pressure from patients and their families. These factors often result in inappropriate prescriptions of antibiotics (5). Surveys and studies have highlighted the need for education and feedback on antimicrobial prescribing. For example, Minen et al. (6) demonstrated the necessity for interventions targeting prescriber behavior in the northeastern United States. Additionally, Cebotarenco and Bush (7) observed that family members, such as mothers, often influence medical decisions, particularly regarding antibiotic prescriptions for children.

Misuse of antibiotics in treating acute respiratory tract infections (ARTIs) and other conditions is a leading contributor to AMR. In a study conducted in China, Tang et al. found that among positive cases of upper respiratory tract infections (URTIs), 81.7% were viral, 11.6% were bacterial, and 6.7% were mixed infections (8). The inability to judiciously differentiate between viral and bacterial infections due to a lack of rapid diagnostic tests has significantly contributed to the misuse of antibiotics (8). Similarly, Collins et al. observed in a United States-based study that 12.3% of cases involving viral gastroenteritis were treated unnecessarily with antibiotics (9).

In low- and middle-income countries (LMICs), including India, over 80% of antibiotic prescriptions for ARTIs are unnecessary (10). Additionally, gastroenteritis and respiratory infections, common outpatient conditions, are frequently mismanaged with antibiotics. In contrast, infections such as urinary tract infections and suppurative/necrotic skin and soft tissue infections, which are bacterial in origin, genuinely require antibiotics for treatment (11, 12). These trends highlight the critical need for targeted interventions to promote the rational use of antibiotics.

Several countries have initiated campaigns to address public misconceptions about antibiotic effectiveness, promote appropriate antibiotic use, and prevent the development of AMR (13–15). In India, the National Action Plan (NAP) for controlling AMR, released in April 2017, outlines strategies to address AMR comprehensively. The first strategy emphasizes improving awareness and understanding of AMR through effective communication, education, and training (16). This approach aligns with the WHO Global Action Plan (GAP) for AMR (17). It underscores the necessity of an organizational or healthcare-system-wide approach to support and monitor the prudent use of antibiotics among the public, physicians, and pharmacists (17).

The public needs to be educated on the ill effects of unnecessary antibiotic use like entering into the post-antibiotic era, increased mortality and morbidity due to drug-resistant infections, obesity with consequences, allergic disorders and inflammatory bowel diseases. They need to be educated that the common respiratory infections and gastroenteritis are most often caused by viruses and few by bacteria. They need to understand that most such infections rarely require antibiotic therapy. This prospective interventional study was hence taken up to create awareness among high school students. This knowledge could help prevent the unnecessary use of antibiotics in restraining:

1. Using unused antibiotics available at home
2. Buying and using the antibiotics directly from the pharmacy
3. Pressurising physicians to prescribe antibiotics
4. Not completing the full course of antibiotics.

## Methodology

**Figure 1:**
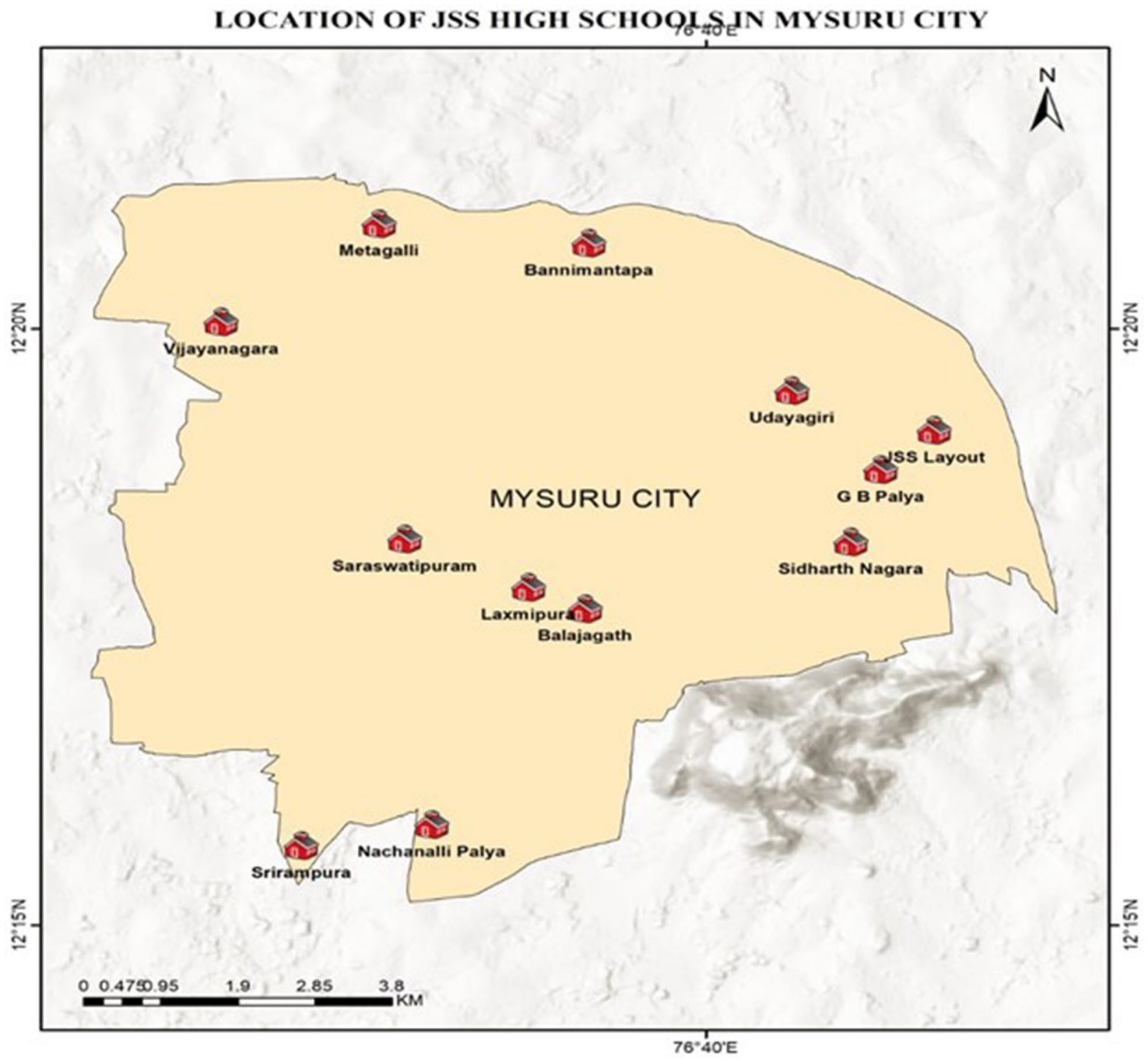
Locations of JSS High School, Mysuru.

*Materials*: High school students from 12 different JSS High Schools, both English and Kannada medium, located in the most thickly populated areas of the city of Mysuru, Karnataka, India, mostly belonging to middle and lower socio-economic strata, constituted the study material. Informed consent from each school was taken before the scientific awareness program. Figure 1 depicts the areas from which different schools were chosen to create awareness, and Table 1 depicts the numbers allotted to each school.

**Table 1:** Depicts the identification numbers allotted to each school.

School name	Numbers allotted to each school	No of students
JSS High School, Bannimantap	1	200
JSS High School, G. B. Palya	2	120
JSS High School, Vijayanagar	3	90
JSS High School, Srirampur	4	120
JSS High School, Lakshmipuram	5	150
JSS High School, Bal Jagath	6	150
JSS High School, JSS Layout	7	80
JSS High School, Saraswathipuram	8	500
JSS High School, Udayagiri	9	140
JSS High School, Metgalli	10	200
JSS High School, Nachinahallipalya	11	200
JSS High School, Siddharth Nagar	12	245
Total		2,195

*Methods*: A total of 2,195 students were included in the study. These students belonged to twelve JSS High Schools spread across various areas of the city of Mysuru. All the students were assembled in the auditorium. For effective management and to ensure an interactive learning experience, the students were divided into fifteen groups (6 to 15 students in each group based on the total strength of each school), with each group having a manageable size for discussions and activities. This division was based on logistical considerations, such as the availability of facilitators, the need for smaller group sizes to promote effective engagement, and the constraints of the physical space available. The pre-test questionnaire was administered to each group for 15 min. The intervention was then conducted through the following:

1. Introduction to four types of microbes (bacteria, viruses, fungi and parasites) and antibiotics through blackboard teaching.
2. Animated video on the ill effects of unnecessary use of antibiotics.
3. Games on how unnecessary antibiotic use can be prevented.

A post-test Questionnaire was administered to assess the effectiveness of the intervention/education.

### Questionnaire

A 30-mark questionnaire was developed in Kannada to evaluate students’ knowledge, attitudes, and practices regarding antibiotic use before and after the intervention. The questionnaire employed a mixed-method approach, combining closed-ended (True/False and multiple-choice) and open-ended (short-answer) questions. This design allowed for both quantitative scoring and qualitative insights into students’ understanding. The same questionnaire was administered pre- and post-intervention to directly measure the impact.

The questionnaire comprised three distinct sections. The first section assessed basic knowledge through two questions: a True/False item testing the understanding of antibiotics’ effectiveness against viral infections, and an open-ended question requiring students to list four ill effects of irrational antibiotic use. The second section presented clinical features of upper/lower respiratory tract infections and gastroenteritis, asking students to identify which bacterial symptoms warranted antibiotic therapy through multiple-choice selection. The third section contained ten True/False statements addressing common misconceptions about antibiotic use for viral infections, potential adverse effects like obesity, and proper usage practices such as completing prescribed courses.

The combination of question types enabled a comprehensive assessment. Administering identical questionnaires before and after the intervention allowed for direct comparison of results. “The intervention included small group discussions, which were conducted to address errors identified in the pre-test. These discussions facilitated collaborative learning and allowed participants to correct misconceptions through peer interactions.” The use of the local language ensured accessibility for all participants.

### Game design

*Rationale for Choosing Specific Games:* The games “Bucketing the Ball” and “Monkeying with Donkey” were selected because they are commonly and frequently played by children across India, ensuring cultural familiarity and participant interest. These games were also chosen for their simplicity and cost-effectiveness, making them ideal for outreach programs. “Monkeying with Donkey,” in particular, was praised for its fun-filled learning, affordability, and ease of setup, making it a practical choice for implementation in resource-limited settings. Their engaging nature made them effective tools for conveying AMR-related messages to young audiences.

#### Bucketing the ball (For Respiratory infections)

*Learning Objective*: Helps in understanding that not all respiratory infections require to be treated with antibiotics, so that unnecessary use of antibiotics can be prevented, and wherever necessary, antibiotics should be administered.

*Method*: Each feature of both viral and bacterial respiratory tract infections was written on individual plastic balls. The above bottles are scattered in four different boxes. These balls were distributed to a set of students. As they played the game, each feature written on the ball was assigned to either a viral or a bacterial cause by the instructors. Accordingly, students were instructed to throw the ball into the two different buckets labelled –one labelled as No antibiotics required (for viral infections) and the other labelled as Antibiotics required (for bacterial infections). Looking at them the other students learnt to differentiate the features of viral and bacterial infections. The next set of students who were made to play had to differentiate the different features by themselves and throw the ball from a distance into the appropriate buckets. Those students who identified the features correctly and appropriately bucketed the ball were rewarded with a pencil.

#### Monkeying with Donkey

*Learning Objective*: The players get to understand who needs to be treated with antibiotics and who does not need to be for gastroenteritis.

*Method:* Waste plastic bottles were labelled with different features of gastroenteritis in different age groups with different health conditions. The above bottles are scattered in four different boxes. These bottles have to be sorted out by the players into two boxes, one labelled -*No antibiotics necessary* and the other labelled- Antibiotics necessary, by dodging the denner who walks in the fixed path as depicted in the picture. If any of the players cannot dodge the touch of the denner, that player replaces the position of the denner and the denner gets into the game. When the players sort out the bottles appropriately, dodging the denner, the denner gets the letter ‘D’ from the word Donkey. Similarly, another group plays this game and after successful completion of the game, the denner gets the letter ‘O’. This game is completed sequentially by different groups till the different Denners get the letters N, K, E and Y. Whoever gets the last letter Y gets the fun tag “Donkey.”

### Expected outcome

These three interventions created

1. Knowledge of what antibiotics are and for what type of infections antibiotics are to be used.
2. Awareness of when not to use antibiotics and
3. Awareness of the practice of not buying antibiotics directly from the pharmacy without a prescription and completing the course of antibiotics.

Pre-test and Post-test were evaluated and tabulated for further analysis on the effectiveness of the intervention/education. The results were graded as follows, taking into consideration the average score of each school: Very poor for 0–5, Poor for >5–10, Moderate for >10–15, Fair for >15–20, Good for >20–25 and Excellent for >25–30. The results were analysed statistically.

*Piloting the Gamified Intervention*: The gamified interventions were piloted during an awareness program conducted at a fair in Suttur, Mysuru, South India. The pilot study targeted primary and high school students, with 30 participants included to evaluate the interventions’ feasibility and effectiveness. Baseline knowledge of antimicrobial resistance (AMR) among the participants was low, with only 30% demonstrating awareness before the intervention. After participating in the games, 85% of the students showed improved understanding of AMR-related concepts. Additionally, 90% of the participants rated the games as highly engaging, with “Monkeying with Donkey” particularly appreciated for its simplicity and entertainment value. The results validated the interventions’ potential for broader application in awareness campaigns.

*Statistical analysis:* Data analysis was carried out using SPSS software version 20.0. A paired t-test was used to compare the pre-test and post-test percentages for each component, as the data were paired, with each group serving as its control.

## Results

The different components assessed pre-test and post-test by innovative games on antibiotics were:

- If they knew the use of antibiotics was only in bacterial infections
- Knowledge of the ill effects of unnecessary antibiotic use.
- Awareness of when not to use and when to use antibiotics in common infections like respiratory tract infections and gastroenteritis.
- Awareness of the use of antibiotics in urinary tract infections and skin and soft tissue infections.
- The practice of buying antibiotics from a pharmacy and completing the course of antibiotics prescribed by the doctors.

Table 2 depicts the five different components assessed pre- and post-test. The knowledge of the effects of antibiotics was the lowest during the pre-test (2.5%). The knowledge of using antibiotics only for bacterial infections was also very low (11.5%). The awareness of when not to use antibiotics and when to use them for common infections, such as respiratory tract infections and gastroenteritis, was also very low (5.1%). The awareness of the use of antibiotics for urinary infection and skin and soft tissue infection was better compared to the first three components (19.6%) in the pre-test. The practice of not buying antibiotics from the pharmacy and completing the antibiotic course was also better compared to the first three components (20.3%) in the pre-test.

**Table 2:** Depicts the five different components assessed pre- and post-test.

Component	Pre-test in %	Pre-test CI	Post-test in %	Post-test CI	*p* value	Effect size
I	11.5	10.17 to 12.83	82.5	80.91 to 84.09	0.03	94.44
II	2.5	1.85 to 3.15	82.5	80.91 to 84.09	0.03	106.97
III	5.1	4.18 to 6.02	96.77	96.03 to 97.51	0.03	122.8
IV	19.6	17.94 to 21.26	90.38	89.15 to 91.61	0.03	94.65
V	20.3	18.62 to 21.98	91.92	90.78 to 93.06	0.03	95.77

After the intervention, the knowledge of the ill effects of antibiotics improved from 2.5 to 82.5% (*p*-value 0.003). The knowledge of using antibiotics only for bacterial infections improved from 11.5 to 82.5% (*p*-value 0.003). The awareness of when not to use antibiotics and when to use them for common infections, such as respiratory tract infections and gastroenteritis, improved from 5.1 to 96.77% (*p*-value 0.003). The awareness of the use of antibiotics for urinary infections and skin and soft tissue infections improved from 19.6 to 90.38% (*p*-value 0.003). The practice of not buying antibiotics from the pharmacy and completing the antibiotic course improved from 20.3 to 91.92% (*p*-value 0.003).

As per the grading described in the methodology, in the pre-test, majority of the schools had poor knowledge and awareness (10/12 schools), and two schools had very poor performance. In the post-test, test majority of the schools had good performance (9/12 schools), two schools had fair performance (2/12), and only one school had an excellent performance. Table 3 depicts the performance of different schools before and after the intervention.

**Table 3:** Depicts the performance of different schools before and after the intervention.

School name	No of students	Pre-test -Mean ± SD and (Grading)	Pre-test 95% CI	Post-test -Mean ± SD and (Grading)	Post-test 95% CI	*p*-value
School-01	200	6.93 ± 2.15 (Poor)	6.63–7.23	21.73 ± 3.34 (Good)	21.27–22.19	8.95 × 10^−152^
School-02	120	5.46 ± 3.11 (Poor)	4.90–6.02	20.66 ± 4.43 (Good)	19.87–21.45	3.01 × 10^−74^
School-03	90	7.26 ± 2.49 (Poor)	6.74–7.78	22.4 ± 4.45 (Good)	21.48–23.32	1.17 × 10^−63^
School-04	120	7 ± 2.2 (Poor)	6.60–7.40	20 ± 4.17 (Fair)	19.25–20.75	1.22 × 10^−78^
School-05	150	4.33 ± 2.87 (Very Poor)	3.87–4.79	21.86 ± 3.94 (Good)	21.23–22.49	4.42 × 10^−103^
School-06	150	6.66 ± 2.35 (Poor)	6.28–7.04	26.46 ± 3.39(Excellent)	25.92–27.00	3.95 × 10^−128^
School-07	80	4.4 ± 3.04 (Very Poor)	3.73–5.07	23.8 ± 3.52 (Good)	23.02–24.58	6.71 × 10^−65^
School-08	500	5.53 ± 3.52 (Poor)	5.22–5.84	19.73 ± 3.78 (Fair)	19.40–20.06	1.14 × 10^−306^
School-09	140	6.86 ± 2.61 (Poor)	6.43–7.29	23.46 ± 3.15 (Good)	22.94–23.98	2.06 × 10^−110^
School-10	200	6.66 ± 2.41 (Poor)	6.33–6.99	24.2 ± 4.07 (Good)	23.64–24.76	1.62 × 10^−157^
School-11	200	6.26 ± 2.71 (Poor)	5.88–6.64	22.6 ± 4.03 (Good)	22.04–23.16	5.32 × 10^−141^
School-12	245	6.13 ± 2.35 (Poor)	5.83–6.43	25 ± 2.75 (Good)	24.65–25.35	3.26 × 10^−226^

## Discussion

In this study, the knowledge of the use of antibiotics for bacterial infections improved from 11.5 to 82.5%. In the study conducted by Azevedo et al. (5) found that the knowledge of the correct use of antibiotics for bacterial infections, rather than viral, rose from 43 to 76% after the teaching activity. Similarly, in our study, the awareness of when not to use and when to use antibiotics for respiratory tract infections and gastroenteritis improved from 5.1 to 96.77%. The awareness of the use of antibiotics for urinary infections and skin and soft tissue infections improved from 19.6 to 90.38%. Furthermore, the practice of not buying antibiotics from the pharmacy and completing the antibiotic course improved from 20.3 to 91.92%. There is a lack of studies similar to ours, and hence, much of the literature was not available to correlate.

In our study, out of twelve schools, one school had an excellent performance, while nine schools had a good performance, and two schools had a fair performance post-intervention. After the intervention, the knowledge of the ill effects of antibiotics improved from 2.5 to 82.5% (*p*-value 0.03). In a study conducted by Azevedo et al. (5) the knowledge of the risk of resistance to antibiotics from their irrational use rose from 48 to 74% after the teaching activity.

During the study, we observed that wherever the teachers had a good commitment to getting the students educated, the post-test results of these students were much better. This indicates that training the school teachers on the rational use of antibiotics would help their students to learn about this issue better and help improve the awareness among the school children. However, it remains unclear whether the knowledge gains observed in this study will be retained in the long term. A follow-up assessment after a few months would provide valuable insight into whether these improvements persist.

Studies from countries like Kuwait, Arab countries, Tanzania, Malaysia, and Ethiopia have concluded that the study population have low knowledge and awareness of antibiotic use and AMR. Many misconceptions prevail on the use of antibiotics for viral infections like the flu and all types of fever. For these infections, people demand antibiotics from the doctors, and doctors prescribe antibiotics to meet the patient’s expectations. The patients are found to discontinue the antibiotics once they feel symptomatically better. These studies have concluded that dissemination of awareness on the rational use of antibiotics is essential to fight against rising antimicrobial resistance (7, 8). In the Indian context, Thoufiq et al. conducted a cross-sectional study in Tamil Nadu that reported the wrong practice of self-medication with antibiotics and identified gaps in awareness among the population. Similarly, Chandy et al. conducted a qualitative study in Tamil Nadu, observing patterns of antibiotic use, and emphasized the critical role of antibiotic stewardship interventions to combat AMR in India (18, 19).

In contradiction to all the above studies, a study from Romania has concluded that the study participants had adequate knowledge of antibiotics and their use. There is an urgent need to raise awareness of antimicrobial resistance and the rational use of antibiotics in the wider population. It was observed that even well-educated adults did not have a clear idea that antibiotics can be used only in bacterial infections. Education through television, radio, YouTube lectures, and documentary movies can be used to reach out to the larger population. Educating people using social media like WhatsApp, Facebook, Instagram, LinkedIn, Twitter, etc., can be exploited for this purpose. Many other health awareness days, like World Diabetes Day, World Tuberculosis Day, World Heart Day, etc., have wider publicity in the media, but the World Antibiotic Awareness Week lacks any kind of attention. Hence, the World Antibiotic Awareness Week should be utilized more widely to increase the awareness (20–22).

With the rising incidence of diabetes and immunocompromised states (cancer chemotherapy, HIV-AIDS, immunosuppressive therapy in transplant patients, and autoimmune diseases) across the globe, antibiotics need to be preserved to treat infections in these patients (23). Antibiotics are not only used to treat infections but also used prophylactically in the above-said immunocompromised conditions to save lives.

*Limitations of the study*: While this study demonstrates the effectiveness of gamified education in improving knowledge, attitudes, and practices related to antibiotic use, several limitations should be acknowledged.

- The study design did not include a control group, limiting the ability to conclusively attribute observed improvements solely to the intervention.
- The post-intervention evaluation was conducted immediately after the intervention, with no long-term follow-up to assess the sustainability of knowledge retention and behavioural changes.
- The study was conducted among high school students in a single region (Mysuru, India), and findings may not be generalizable to other populations, age groups, or cultural contexts.
- Pre and post-test responses may have been influenced by social desirability bias or peer/teacher pressure, potentially overestimating the intervention’s impact.
- The effectiveness of gamification may vary depending on student engagement levels, and the selected games might not cater equally to all learning preferences.

*Scalability of the intervention*: The scalability of this intervention has already been demonstrated through its implementation in diverse settings and among varied demographic groups. We conducted the same intervention in Suttur, Mysuru, South India, targeting people of all age groups, highlighting its adaptability to different age demographics. Additionally, it was successfully carried out at the Rotary Club in Mysuru, focusing on adults aged 35 to 60 years, and at the Ladies Club in Mysuru, specifically targeting women aged 30 to 50 years. The intervention at the Ladies Club was particularly significant, as it aimed to engage mothers, recognising their crucial role in influencing medical decisions regarding antibiotic prescriptions for their children.

Furthermore, during Antimicrobial Resistance Awareness Week, we extended our outreach by conducting radio talks to educate the general public, ensuring that we reached a broader audience. By tailoring the content and delivery methods to suit the specific needs and cultural contexts of each group, we demonstrated that the intervention is flexible and scalable. These efforts underscore its potential to be adapted for various cultural and socioeconomic contexts, effectively addressing the needs of multiple stakeholders involved in antibiotic use.

## Conclusion

The knowledge, awareness and practice of rational use of antibiotics are very poor among the general public. With rising AMR and avoiding entering to post-antibiotic era, the need of the hour is to create awareness on the rational use of antibiotics not only among healthcare providers but also among pharmacists and the general public. Greater publicity is required on the rational use of antibiotics and antimicrobial resistance through all types of media. The World Antibiotic Awareness Week should become a national agenda to raise awareness. Innovative gamified interventions create better and long-lasting awareness of this burning global issue.

## Data Availability

The original contributions presented in the study are included in the article/Supplementary material, further inquiries can be directed to the corresponding author.
